# Comparative effectiveness of acupuncture and pharmacological interventions in treating diabetic stroke: A protocol for a systematic review and network meta-analysis

**DOI:** 10.1097/MD.0000000000031823

**Published:** 2022-11-18

**Authors:** Ao Zhang, Fangda Han, Chunli Piao

**Affiliations:** a Nanjing University of Chinese Medicine, Nanjing, China; b Guangzhou University of Chinese Medicine, Guangzhou, China; c Shenzhen Hospital of Guangzhou University of Chinese Medicine, Shenzhen, China; d Shenzhen Hospital of Guangzhou University of Chinese Medicine, Shenzhen, China.

**Keywords:** acupuncture, diabetes, network meta-analysis, pharmacological interventions, randomized controlled trial, stroke, systematic review

## Abstract

There is a close and causative correlation between stroke and diabetes, and the complication of the 2 diseases seriously harms human health and currently becomes a topic of clinical importance. To date, the common methods of treating diabetic stroke include acupuncture and pharmacological interventions. However, there is no high-quality or direct evidence of their comparative effectiveness. This review aims to provide a network meta-analysis to compare the efficacy of acupuncture and pharmacological interventions in treating diabetic stroke.

**Methods:**

Databases such as PubMed, Cochrane Central Register of Controlled Trials, EMBASE, China National Knowledge Infrastructure, China Biology Medicine Disc will be searched for relevant randomized controlled trials to obtain literatures on the treatment of diabetic stroke, and clinical randomized controlled trials will be screened out from their inception to December 30, 2022. The participant intervention comparator outcomes of this study are as flowing: P, patients with diabetic stroke; I, acupuncture and pharmacological interventions; C, no treatment, pharmacological placebo, or sham acupuncture groups; O, primary outcome will be blood glucose levels, glycosylated hemoglobin levels, and the rate of stroke recurrence; secondary outcomes will include fasting and post-load blood glucose levels, cholesterol, triglycerides, and quality of life scale scores. Cochrane Risk of Bias Tool will be used in assessing literature’s quality. Review Manager software 5.3 and Stata 15.1 will be used in data analysis.

**Result:**

This systematic review and network meta-analysis will provide evidence of the efficacy of different therapeutic methods in treating diabetic stroke, to show which forms of therapy are more commonly used with higher effectiveness.

**Conclusion:**

The results will systematically provide suggestions for medical practitioners to choose the effective, time-saving and economical therapeutic strategy for diabetic stroke.

## 1. Introduction

As the second leading cause of death and the leading cause of disability, stroke seriously harms human’s health globally.^[1]^ Diabetes, as a chronic, lifelong, severe metabolic health problem characterized by hyperglycemia, is a well-established independent risk factor for stroke and is associated with increased disability and mortality.^[2]^ There is a close and causative correlation of diabetes and stroke. Diabetes leads to a series of pathological changes, including vascular endothelial dysfunction, increased early age arterial stiffness, systemic inflammation, and thickening of the capillary basal membrane, which raise the risk of stroke, exacerbates neurological deficits after stroke and aggravates stroke pathology.^[3]^ Studies have shown that on a population level, diabetes may be responsible for 8% of first ischemic strokes and doubles their risk for recurrence.^[4]^ For example, type 2 diabetes is associated with a 2.5-times increased risk of ischemic stroke, a 1.5-times increased risk of hemorrhagic stroke.^[5]^ Meanwhile, stroke is increasingly being recognized as a clinically important complication of diabetes.^[6]^ Both stroke and diabetes are increasingly common conditions that contribute to human morbidity and mortality, thereby placing a serious burden on society globally.^[7]^

Patients with comorbidities of diabetes and stroke face more risks than those who suffer from only one of the diseases; studies have proven that diabetes is associated with poorer stroke outcomes and higher post stroke mortality.^[8,9]^ For instance, thrombolysis may not be as effective among diabetic stroke patients as in stroke patients without diabetes; meanwhile, diabetes increases the likelihood of stroke recurrence.^[10]^ At present, the common therapeutic methods for treating diabetic stroke include strict glycemic control, anticoagulant therapy, blood pressure-lowering therapy, lipid-lowering therapy, brain edema control and so on. Nevertheless, there are more or less problems with these treatments, such as limited efficacy, serious adverse reactions, and high cost.^[11,12]^

Acupuncture, as a form of alternative medical treatment based on the theories of traditional Chinese medicine, has been commonly used to treat the symptoms related to diabetes and stroke in addition to the above pharmacological interventions.^[13]^ There are several studies have reported the therapeutic effects of acupuncture in treating diabetic stroke; in clinical practice, acupuncture has been identified as an important method with promising results in regulating glycemic levels, decreasing the rate of stroke recurrence, repairing motor function, and improving life quality, with no apparent adverse reactions.^[14–16]^ Acupuncture appears to be an attractive alternative to pharmacological intervention as a first-line treatment due to its comparatively fewer and less severe side-effects.

Despite both acupuncture intervention and pharmacological intervention being commonly used in treating diabetic stroke, few studies have directly compared them and the previous systematic reviews only evaluated the treatment of diabetic stroke with acupuncture intervention or pharmacological intervention separately. This study aims to compare the efficacy of acupuncture and pharmacological interventions in treating diabetic stroke through a network meta-analysis; acupuncture interventions and pharmacological interventions will be ranked in order to determine the most effective methods, which will provide evidence for choosing certain therapeutic strategies for diabetic stroke.

## 2. Method

This systematic review protocol has been registered on PROSPERO with number CRD42022366474(https://www.crd.york.ac.uk/PROSPERO/display_record.php?RecordID=366474). This protocol will be reported according to the Preferred Reporting Items for Systematic Reviews and Meta-Analyses protocols (PRISMA)^[17]^ and this network meta-analysis will be conducted and reported in accordance with PRISMA extension version (PRISMA-NMA).^[18]^ We will also apply the International Society for Pharmacoeconomics and Outcomes Research Indirect Treatment Comparison/Network Meta-Analysis Study Questionnaire to Assess Relevance and Credibility to Inform Health Care Decision-Making to our study to aid the interpretation of clinicians and other healthcare decision-makers.^[19]^

### 2.1. Eligibility criteria

#### 2.1.1. Population.

People diagnosed with both stroke and diabetes will participate without considering any information related to their age, race, nationality, education, sex, or economic status.

#### 2.1.2. Interventions.

The systematic review will focus on acupuncture and pharmacological interventions; among which, the pharmacological therapy options are derived from the first-line treatments in the recent guidelines and consensus for diabetic stroke, including antidiabetic medications (e.g., including biguanides, insulin, glitazones, sulfonylureas, glucagon-like peptide 1 receptor agonists, sodium-glucose co-transporter-2 inhibitors, etc), antihypertension medications (e.g., angiotensin receptor blocker angiotensin-converting enzyme inhibitors, statins, etc), anticoagulant medications (such as acetylsalicylic acid).^[20–22]^

#### 2.1.3. Comparators.

There are a number of types of comparator conditions which will be eligible for inclusion in the network of evidence. The types of control conditions may include no treatment, pharmacological placebo, or sham acupuncture groups. Furthermore, different types of acupuncture interventions and pharmacological therapies may also be directly compared.

#### 2.1.4. Outcomes.

The primary outcome measures will be the levels of blood glucose and glycosylated hemoglobin A1c (HbA1c), and the rate of stroke recurrence.

The secondary outcomes of this review will include other clinical parameters associated with stroke and diabetes, including fasting and post-load blood glucose levels, cholesterol, triglyceride levels, and quality of life scale scores.

#### 2.1.5. Study designs.

The systematic review will only include randomized controlled trials. These trials must have a sample size of at least 20 participants per condition and must involve interventions delivered for a minimum of 4 weeks. To reduce heterogeneity, crossover trials will be excluded. Studies making within-class comparisons only (e.g., ordinary acupuncture versus electroacupuncture) will also be excluded. Finally, studies must be reported in English or in Chinese.

### 2.2. Information sources and search strategy

We will search PubMed, Cochrane Central Register of Controlled Trials, EMBASE, China National Knowledge Infrastructure, China Biology Medicine Disc from inception to December 30, 2022 with the language restriction of English and Chinese. Randomized controlled trials (RCTs) that exhibited the effective therapies of diabetic stroke will be selected. The search keywords contain stroke (e.g., cerebral infarction, cerebral hemorrhage), diabetes (e.g., diabetes mellitus, diabetes), acupuncture (e.g., electroacupuncture, moxibustion, cupping) and drugs for diabetic stroke (e.g., insulin, thiazolidinediones, aspirin, simvastatin). The search strategy for PubMed is shown in Table 1, and other electronic databases will be searched with the same strategy.

**Table 1:** Search strategy for PubMed database.

Number	Search terms
#1	“Stroke” [Mesh] OR “Cerebral Infarction” [Mesh] OR “Brain Infarction” [Mesh] OR “Brain Ischemia” [Mesh] OR “Cerebral Infarction” [Mesh] OR “Brain Infarction” [Mesh] OR “Brain Ischemia” [Mesh] OR “Cerebral Hemorrhage” [Mesh] OR “Intracranial Hemorrhages” [Mesh]
#2	“Stroke” [Title/Abstract] OR “Cerebral Ischemia” [Title/Abstract] OR “Cerebral Ischaemia” [Title/Abstract] OR “Brain Ischaemia” [Title/Abstract] OR “Ischemic Brain” [Title/Abstract] OR “Ischaemic Brain” [Title/Abstract] OR “Cerebral Infarct” [Title/Abstract] OR “Brain Infarct” [Title/Abstract] OR “Cerebral Artery Infarct” [Title/Abstract] OR “Cerebral Artery Infarction” [Title/Abstract] OR “Cerebral Circulation Infarction” [Title/Abstract] OR “Cerebral Artery Thrombosis” [Title/Abstract] OR “Cerebral Artery Thrombotic Infarction” [Title/Abstract] OR “Cerebral Artery Embolic Infarction” [Title/Abstract] OR “Brain Venous Infarction” [Title/Abstract] OR “Cerebral Hemorrhage” [Title/Abstract] OR “Intracranial Hemorrhage” [Title/Abstract] OR “Hemorrhagic Stroke” [Title/Abstract]
#3	#1 OR #2
#4	“Diabetes Mellitus, Type 2” [Mesh] OR “Type 2 Diabetes” [Title/Abstract] OR “Diabetic” [Title/Abstract] OR “Diabetes” [Title/Abstract] OR “Mellitus” [Title/Abstract] OR “Hyperglycemia” [Title/Abstract]
#5	“Acupuncture” [Mesh] OR “Chinese External Medicine” [Title/Abstract] OR “Electroacupuncture” [Title/Abstract] OR “Fire Needle” [Title/Abstract] OR “ Needling” [Title/Abstract]
#6	“Insulin” [Mesh] OR “Metformin” [Mesh] OR “Thiazolidinediones” [Mesh] OR “Sulfonylurea Compounds” [Mesh] OR “Aspirin” [Mesh] OR “Clopidogrel” [Mesh] OR “Rosiglitazone” [Mesh] OR “Pioglitazone” [Mesh] OR “GLP-1 receptor agonists” [Title/Abstract] OR “Simvastatin” [Mesh] OR “Semaglutide” [Mesh] OR “SGLTi” [Title/Abstract] OR “Empagliflozin” [Mesh] OR “Dapagliflozin” [Mesh] OR “Liraglutide” [Mesh] OR “rGLP-1 protein” [Mesh] OR “Dulaglutide” [Mesh] OR “Captopril” [Mesh] OR “Lisinopril” [Mesh]
#7	#5 OR #6
#8	“Randomized Controlled Trial” [Title/Abstract] OR “Controlled Clinical Trial” [Title/Abstract] OR “Clinical Trial” [Title/Abstract] OR “Clinical Trial” [Publication Type] OR “RCT” [Title/Abstract]
#9	#3 AND #4 AND #7 AND #8

### 2.3. Data collection and analysis

#### 2.3.1. Study selection.

**Figure 1.**
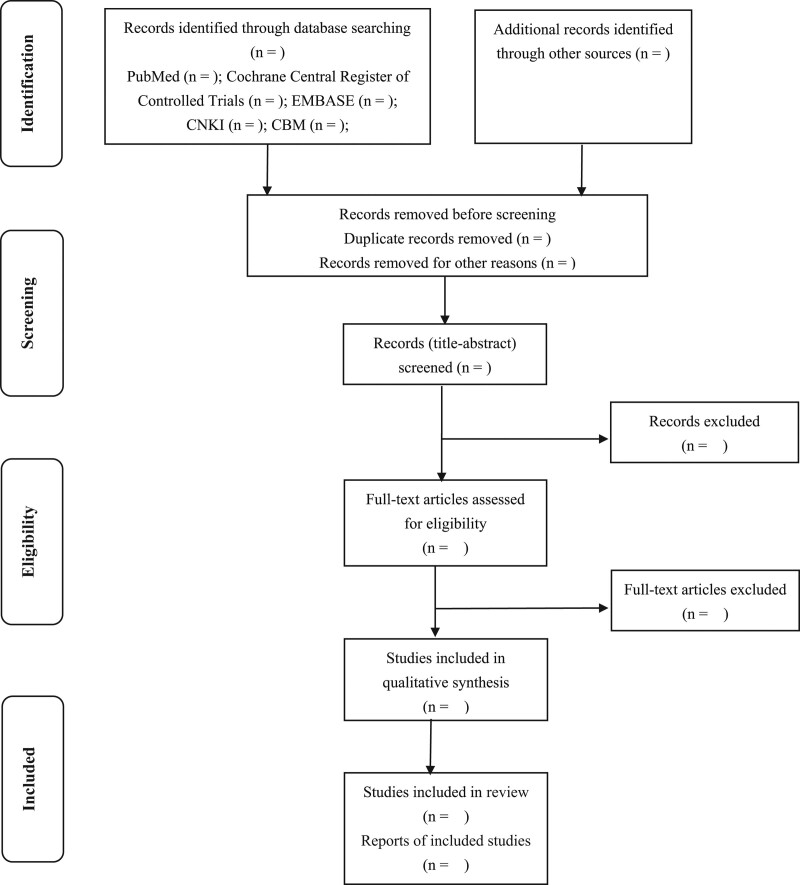
Flow diagram of the study selection process. Adapted from Preferred Reporting Items for Systematic reviews and Meta-Analysis Protocols.

All reviewers will receive professional training to understand the objective and process of the review before the selection of studies. Literature search results will be imported into ENDNOTE X8 software. The duplicates will be removed. For studies that have been updated, the older one will be excluded, or can be used as supplementary data in further research. Titles and abstracts will be screened independently by 2 reviewers (FDH and ZA). Full texts will be obtained for eligible studies and will be screened independently (FDH and ZA). Discrepancies will be resolved through discussion, or by consulting a third reviewer (CLP). The procedures of study selection will be performed in accordance with the Preferred Reporting Items for Systematic reviews and Meta-Analysis flow chart (as shown in Fig. 1).

#### 2.3.2. Data extraction and analysis.

Two reviewers (FDH and ZA) will establish a sheet using Microsoft Excel 2016, pilot and refine this form using 10 initial studies. After the form has been developed, the 2 reviewers will extract data from the text and figure/table independently, including: general information (e.g., author, year of publication, country where the study was conducted, study period, original inclusion criteria); participants (e.g., age, gender, sample size); details about intervention (e.g., drug name, dosage, administration method, duration of study, follow-up time, acupuncture parameters, acupuncture points); study design (e.g., randomization, blinding and allocation concealment); outcomes and adverse reactions.

Discrepancies will be identified and resolved through discussion (with a third participant where necessary), or by consulting a third reviewer (CLP). We first try to extract numerical data from tables, text or figures. If these are not reported, we will extract data from graphs using digital ruler software. In case data is not reported or unclear, we will attempt to contact authors by e-mail (max. 2 attempts).

Review Manager software 5.3 and Stata 15.1 will be used in data analysis.^[24]^ For dichotomous data, a risk ratio with 95% confidence intervals will be used for analysis. For continuous data, a mean difference or a standard mean difference with 95% confidence intervals will be used for analysis.

#### 2.3.3. Classification of arms.

Classification of arms will be carried out at the data extraction stage. The arms of each trial will be classified according to the type of acupuncture intervention, type of drug class administered or type of control condition employed, where applicable. The classes of drug included in this study are those used as the first-line treatment, as indicated by the guidelines and consensus for diabetic stroke.^[20–22]^ The acupuncture interventions will be classified according to the different methods in clinical practice, which will include ordinary acupuncture, electro-acupuncture, auricular acupuncture, acupuncture injection, moxibustion, bloodletting, cupping, fire needle, and plum blossom needle; combinations of the any of the previously listed types of acupuncture intervention may be included. Control conditions will be classified as active or passive. Passive control conditions are those where no intervention is applied, while active control conditions involve some intervention (e.g., placebo).

A network diagram will be generated to visualize the evidence available for analysis, both in terms of possible pairwise comparisons and the volume of evidence underlying each of these comparisons. The reference node will contain active control conditions. Different combinations of interventions will be included as separate nodes. A network diagram will be generated to visualize the evidence available for analysis, both in terms of possible pairwise comparisons and the volume of evidence underlying each of these comparisons.^[23]^ The reference node will contain active control conditions. Different combinations of interventions will be included as separate nodes.

#### 2.3.4. Risk of bias.

For studies which are passed through the full-text screening stage, risk of bias will be assessed independently by 2 reviewers (FDH and ZA) using the standard Cochrane risk of bias tool in Covidence which assesses sequence generation and allocation concealment, blinding of participants and personnel, blinding of outcome assessors and incompleteness of the outcome data and whether reporting appears to be selective.^[24]^ Discrepancies will be identified and resolved by consulting a third reviewer (CLP).

#### 2.3.5. Assessment of transitivity.

A table of trial characteristics which may act as effect modifiers will be compiled from the data collected (as specified above) to aid in the assessment of the assumption of transitivity. Potential effect modifiers include total study duration, setting, age distribution, gender distribution, ethnicity distribution, patient comorbidity history and past/present medication use.^[25,26]^

### 2.4. Data synthesis

The main objective of this data synthesis is to compare the effectiveness of different interventions focused on acupuncture or pharmacological interventions. Network meta-analysis is useful for achieving this objective because it allows indirect estimates to be computed where little direct evidence exists. Few studies have directly compared acupuncture interventions to pharmacological interventions. Studies will be pooled according to the trial arm classifications noted above. If quantitatively pooling the study results is not possible, the findings of the systematic review will be described narratively.

#### 2.4.1. Pairwise meta-analysis.

Where head-to-head data is available, exploratory pairwise meta-analyses will be conducted. These will be run using a random-effects model. The individual and pooled effect sizes will be visualized using Forrest plots. Funnel plots and Egger’s test will be employed to examine publication bias and the effects of small studies.^[27]^

#### 2.4.2. Network meta-analysis.

If the assumption of transitivity is deemed to be met, random-effects Network meta-analyses will be conducted within a Bayesian framework using vague priors. These analyses will be carried out in line with the framework set out by Dias et al.^[28]^ Estimates of the pairwise comparison of each intervention in the network will be presented in tables in the final manuscript, as will rankings demonstrating the probability of each intervention producing the best outcome. These rankings will be presented with mean ranks, 95% credible intervals and the surface under the cumulative ranking curve. Convergence will be assessed by checking if the Gelman-Rubin statistic is less than 1.1.^[29,30]^

#### 2.4.3. Assessment of inconsistency, heterogeneity and quality of the evidence.

Statistical heterogeneity will be assessed for each pairwise meta-analysis using the *I*^2^ and Tau^2^ statistics in line with the Cochrane guidelines. Since the included studies are likely to consist of a mixture of 2-arm and multi-arm studies, it is necessary to consider design inconsistency as well as loop inconsistency. This will be achieved by applying a design-by-treatment interaction model. If inconsistency is indicated in the network, any closed loops within the network will be assessed.^[31]^

The quality of the evidence used in this study will be assessed using the GRADE 4-step approach for rating the quality of treatment effect estimates from network meta-analysis.^[32]^

#### 2.4.4. Additional analyses.

Exploratory analyses will be carried out, where there is a sufficient amount of information available to do so. These analyses will focus on the covariates the age distribution, gender distribution and ethnicity distribution. Network meta-regressions will be conducted to individually examine the influence of these covariates on effect size estimates. Finally, sensitivity analyses will be conducted to assess the influence of the use of specific treatments in the network rather than classes and trial quality.

## 3. Discussion

According to statistics, by 2030, the number of first-time stroke patients will reach 23 million and the number of deaths caused by stroke will reach 7.8 million.^[33]^ The prevalence of diabetes in developed and developing countries has risen substantially, and type 2 diabetes is estimated to affect 380 million people worldwide by 2025.^[34]^ To address the present and future challenges, it is important to find rational and effective medical options for diabetic stroke.

Previous studies have shown that acupuncture interventions can improve the condition of diabetic stroke by affecting metabolism, repairing endothelial cells, and protecting the internal structure of neurons, and so on.^[35–39]^ However, more evidence-based information is needed for further clinical promotion and application of acupuncture interventions for diabetic stroke. We hope that through this study, evidence on the efficacy of acupuncture and pharmacological interventions for diabetic stroke will be found, and the rankings will be provided by using a network meta-analysis. This study aims to provide reliable evidence-based information for improving the development of clinical therapeutic strategies for endocrinological and cerebrovascular diseases, and bring more hope to patients with diabetic stroke.

## Author contributions

Ao Zhang tested the feasibility of the study, contributed to the development of the selection criteria, Fangda Han designed this research and drafted the manuscript, and the risk of bias assessment strategy. Chunli Piao read, provided feedback and approved the final manuscript. All authors approved the final version of the manuscript.

**Conceptualization:** Ao Zhang, Fangda Han.

**Project administration:** Ao Zhang.

**Writing – original draft:** Ao Zhang, Fangda Han.

**Writing – review & editing:** Chunli Piao.
